# Epidemiology of intussusception among infants in Togo, 2015-2018

**DOI:** 10.11604/pamj.supp.2021.39.1.21343

**Published:** 2021-07-29

**Authors:** Enyonam Tsolenyanu, Komlatsè Akakpo-Numado, Djatougbe Eliane Akolly, Jason Mathiu Mwenda, Jacqueline Tate, Amevegbe Boko, Dadja Landoh, Komlan Gnassingne, Yawo Atakouma, Umesh Parashar

**Affiliations:** 1 Department of Paediatrics, Medical School of Lome, Togo, West Africa,; 2 Ministry of Health, Togo,; 3 Department of Paediatrics Surgery, Medical School of Lome, Togo, West Africa,; 4 The World Health Organization, Regional Office for Africa, Brazzaville, Congo,; 5 National Center for Immunization and Respiratory Diseases, Centers for Disease Control and Prevention, Atlanta, USA,; 6 The World Health Organization, Country Office, Togo

**Keywords:** Intussusception, infant, Togo, Sub-saharan Africa

## Abstract

**Introduction:**

intussusception is the leading cause of bowel obstruction in infants and young children. We describe the epidemiology and diagnostic and treatment characteristics of intussusception among Togolese infants over a 4-year period.

**Methods:**

we implemented active surveillance among infants younger than 1 year of age admitted with intussusception from 2015 to 2018 at Sylvanus Olympio Teaching Hospital and in 2018 at Campus Teaching Hospital. Brighton Collaboration Level 1 case definition criteria were used to confirm the diagnosis of intussusception.

**Results:**

during four years, 41 cases of intussusception, with an annual range of 8 to 14 cases (median: 10) were reported; and the highest number of cases (89%) was enrolled at Sylvanus Olympio teaching hospital. Intussusception was uncommon in the first 2 months of life, peaked from 5 to 7 months old (63%), with male predominance (63%), and showed no significant seasonality. One third of cases (34%) were transferred to the sentinel surveillance site from another health facility; and the median delay in seeking care was 4 days (range: 0-11) with ≥ 48-hour delay in 59% of cases. Clinical symptoms, ultrasound and surgery were combined to diagnose intussusception in all the cases (100%). The treatment was exclusively surgical, and intestinal resection was common (28/41, 68%). A high case fatality rate (23%) was observed and the average length of hospital stay was 10 days (range: 1-23).

**Conclusion:**

active surveillance for intussusception in Togo has highlighted exclusive use of surgical therapy; often associated to an intestinal resection with a very high case fatality rate.

## Introduction

Intussusception (IS) is defined as invagination of one segment of intestine within a more distal segment [1]. It is the leading cause of intestinal obstruction in children and infants, occurring without any identifiable cause in 90% of cases [2]. Boys are often more affected than girls; and delay in treatment may result death [3]. Globally, the rate of naturally occurring IS in infants younger than 1 year of age was estimated to be 74 cases per 100 000 infants, with a peak incidence among 5-7 month old infants [4]. Data are available on IS worldwide before rotavirus vaccine introduction in immunization schedules [4-10]; but few studies were published from the African region [11-13]. The incidence rate of IS was 56 per 100 000 among infants younger than 1 year of age in South Africa. This incidence ranged from 31 per 100 000 in South Africa to 60 per 100 000 in Zambia among children younger than 2 years of age [4, 14]. In Togo, at least two studies have been published prior to rotavirus vaccine introduction; both at Sylvanus Olympio teaching hospital; but none of them was specifically about epidemiology of the disease. The first was a 6-year retrospective IS study included 37 infants aged 2-13 month; and the second was a cross-sectional prospective study over a 2-year period that included 15 young children aged 4-42 months. Boys were predominant in both studies. The average delay in seeking care was 4 days; with 66% of cases treated after > 48 hours. Surgery was the treatment method in over 94% of cases; with a range of 22 to 27% case fatality rate [15, 16].

Vaccines are now available against rotavirus, the leading cause of severe diarrhea associated to death among children worldwide [17]. A previously available rotavirus vaccine was associated with IS [18, 19]; thus large clinical trials (~70,000 infants) were conducted in US, Europe, and South America for the currently available vaccines (Rotarix® and RotaTeq®). These pre-licensure data found no evidence of an association between IS and rotavirus vaccines [20, 21]. Clinical trials from two newer rotavirus vaccines (Rotavac® and Rotasiil®), which were recently pre-qualified for use by the World Health Organization, were not powered to assess an association of vaccination with IS [22-24]. However, post licensure data from middle and high-income countries such as USA, Mexico, Australia and Brazil suggest a minimally increased IS risk in vaccinated infants [25-33]. Nevertheless, given the magnitude of declines in rotavirus disease and associated mortality, compared with this small increase in the risk of IS, the benefits of rotavirus vaccination outweigh the small increase risk of IS [34-36]. Further, active surveillance for IS in seven lower-income sub-Saharan countries has shown that the risk of IS after administration of monovalent human rotavirus vaccine (Rotarix) was not higher than the background risk of IS [37]. According to the recommendation from the World Health Organization [38], Togo introduced the monovalent human rotavirus vaccine -Rotarix®- in its routine immunization schedule in June 2014 (first dose at 6 weeks old and second dose at 10 weeks old). Evidence of early impact of the vaccine on diarrheal disease magnitude among Togolese children has been observed [39-41]. The current study describes the epidemiology and diagnostic and treatment characteristics of intussusception among Togolese infants.

## Methods

### Study design

We conducted active surveillance for IS among infants younger than 1 year of age at Sylvanus Olympio Teaching Hospital from January 2015 through December 2018 and at Campus Teaching Hospital from January to December 2018. All infants in this age group that were admitted to one of these surveillance health facilities with a diagnosis of IS during the surveillance period were enrolled. All surgeons in the surveillance health facilities received standardized training on inclusion criteria for IS cases. Case investigation forms were available at each surveillance health facility. One paediatric surgeon coordinated IS surveillance activities. Periodic visits and phone calls were made to surgeons to encourage reporting of cases and to address likely concerns. Additionally, periodic review of surgical and ultrasound registers was conducted to ensure the thoroughness of reporting.

### Study sites and population under surveillance

Total population of infants younger than 1 year of age in Togo was more than 280 700; and more than 118 500 (42%) of them lived in the geographical catchment area of the two surveillance health facilities. These health facilities were the main national referral hospitals in Togo; and were both located in Lome, the capital city. They are the only health facilities with paediatric surgeons in their staff. The current IS surveillance coordinator was based at Sylvanus Olympio Teaching Hospital from January 2015 to December 2017 and in January 2018, he moved to Campus Teaching Hospital to inaugurate a paediatric surgical ward at this health facility. No health facilities in the country have the ability to perform enema contrast in children, due to the lack of adequate contrast medium. Often in case of IS, the child was first admitted to pediatric ward; and was only referred to surgery when ultrasound result was in favour of diagnosis.

### Case definition

We used Brighton Collaboration Level 1 case definition criteria for the diagnosis certainty of IS. Confirmation of IS by air and/or liquid contrast enema, and/or ultrasound (with confirmed reduction on subsequent ultrasound or enema), and/or at surgery and/or autopsy is classified as Level 1 [1]. As enema contrast medium was not availability during surveillance period, included cases were those confirmed by ultrasound and/or surgical criteria.

### Data collection and analysis

The main variables collected by the study were gender, dates of birth, symptom onset, admission to first health facility, admission to surveillance health facility, and disposition, diagnosis certainty definition criteria, diagnostic method(s), treatment method, and outcome at discharge. Collected data were analysed by Epi Info 7.

**Disclaimer:** the findings and conclusions in this paper are those of the authors and do not necessarily represent the official position of the World Health Organization or those of Centers for disease control and prevention.

## Results

### Summary of surveillance findings

**Figure 1:**
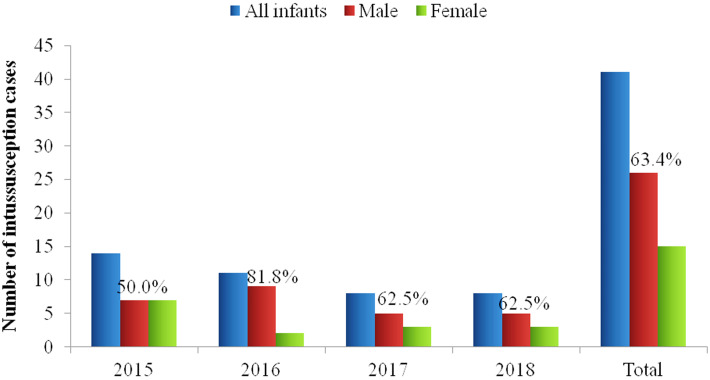
gender distribution of infants younger than 1 year of age with intussusception by hospitalization year, 2015 - 2018, Sylvanus Olympio Teaching Hospital and Campus Teaching Hospital, Lome, Togo; the proportion of intussusception cases in boys is noted

**Figure 2:**
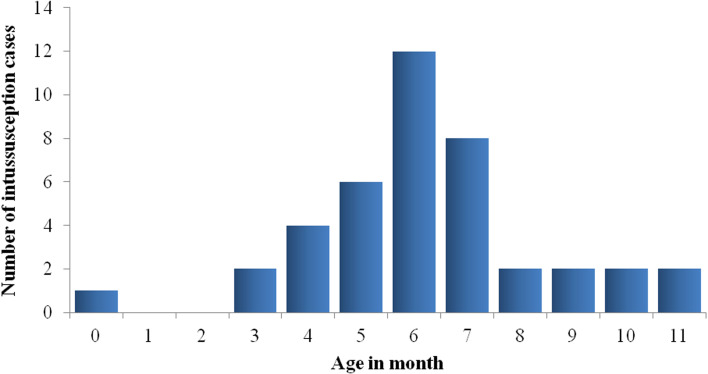
age distributions of infants younger than 1 year of age with intussusception, 2015 - 2018, Sylvanus Olympio Teaching Hospital and Campus Teaching Hospital, Lome, Togo

**Figure 3:**
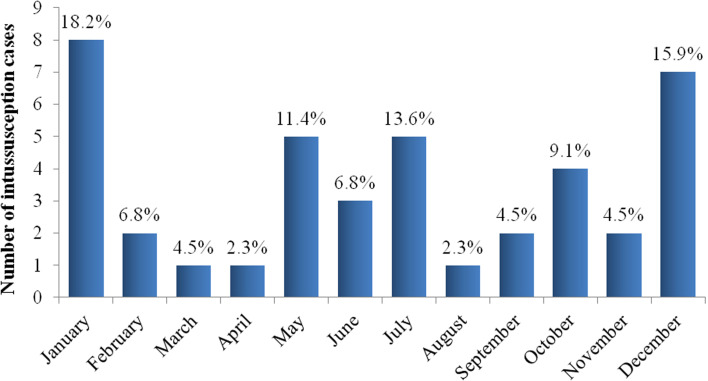
monthly distribution of infants younger than 1 year of age with intussusception, 2015- 2018, Sylvanus Olympio Teaching Hospital and Campus Teaching Hospital, Lome, Togo; the proportion of intussusception cases by month is reported

During IS active surveillance period, 41 cases of IS were reported among infants younger than 1 year of age; 36 (88%) of them were admitted to Sylvanus Olympio National Referral and Teaching Hospital from 2015 to 2018, and 5 (12%) to Campus Teaching Hospital through 2018. A range of 8 to 14 (median: 10) IS cases were reported annually; and 63% (26/41) were boys (Figure 1). A peak of reported cases (26/41, 63%) was observed among 5-7 month old infants (Figure 2). January (19%, 8/41) and December (17%, 7/41) had the highest number of cases (Figure 3).

### Description of diagnostic and treatment characteristics

The diagnostic methods were multiple: clinical symptoms (child admission for acute abdominal pain, vomiting, associated with mucous and bloody stools), ultrasound and surgery in all the cases (100%). Air or liquid enema and autopsy were not performed in any case. Thirty-four percent (14/41) of cases were transferred to the surveillance health facility from another health facility. A range of 0 to 7 (median: 3 days) interval occurred from admission to the first health facility to transfer to the surveillance health facility. The overall care seeking median delay was 4 days (range: 0-11) from symptom onset; and 59% (24/41) of the cases were admitted to the surveillance health facility only 48 hours or more after the symptom onset. All IS cases were treated by surgical operation; with an intestinal resection in 68% (28/41) of cases. Nine cases died for a case fatality rate was 22% (9/41). Data on duration of hospital stay was available in 78% (32/41) of cases, and ranged from 1 to 23 days; with 10-day average length of stay (Table 1).

**Table 1:** overview of intussusception cases among infants younger than 1 year of age at Sylvanus Olympio Teaching Hospital and Campus Teaching Hospital, 2015 - 2018, Lome, Togo

Items	N=41	%
**Surveillance sites**		
Sylvanus Olympio Teaching Hospital (2015-2018)	36	87.8
Campus Teaching Hospital (2018)	5	12.2
**Infant transferred from another health facility**		
Yes	14	34.1
No	27	65.9
**Number of day(s) between admission to first health facility and admission to surveillance health facility**		
0-1 day	6	42.9
2-3 days	6	42.9
4-7 days	2	14.2
Not applicable	27	-
**Number of day(s) between symptoms onset and admission to surveillance health facility**		
0-1 day	17	41.5
2-3 days	12	29.3
4-7 days	11	26.8
>7 days	1	2.4
**Infant meet Brighton level 1 definition criteria for intussusception**		
Yes	41	100.0
No	0	0.0
**Diagnosis of intussusception**		
Clinical symptoms*, ultrasound and surgery	41	100.0
Clinical symptom, ultrasound, enema	0	-
Clinical symptom, ultrasound, autopsy	0	-
**Treatment of intussusception**		
Surgery	41	100.0
Spontaneous reduction	0	0.0
**Intestinal resection**		
Yes	28	68.3
No	13	31.7
**Number of days between admission and disposition from surveillance health facility**		
1-5 days	7	21.9
6-10 days	13	40.6
11-15 days	5	15.6
16-23 days	7	21.9
Missing data	9	-
**Outcome at discharge**		
Discharge home	31	77.5
Death	9	22.5
Missing data	1	-

* child admission for acute abdominal pain, vomiting, associated with mucous and bloody stools.

## Discussion

Our findings compared to available previous data on IS disease in Togo, confirmed also male predominance. Data from previous studies did not allow any comparison for likely change in age distribution [15, 16]. The very small number of intussusception cases identified precluded us from evaluating the possible association between rotavirus vaccine administration and occurrence of IS. Moreover, compared to previous data in Togo, we did not observed any improvement regarding seeking health care delay, treatment method, and case fatality rate; although the problem were already raised since the last two studies published respectively in 2004 and 2012 [15, 16]. Several publications on IS disease had already mentioned this poor management of cases in developing countries, particularly in Africa. Surgery was the common treatment method in Africa and was used in over 76% of cases with highest case fatality rates up to 34% [4, 11-14, 36]. Efforts can be made to improve IS case management in Africa as it is in high-income countries. In high-income countries, seeking health care delay is shortened, non-invasive methods are used to reduce IS in most cases, and surgery is required in less than 30% of cases resulting in dramatically low case fatality rates less than 1% [4]. In some middle-income countries even if surgery is still often use, they are improving in reduction of case fatality rate [4, 6-9]. This example can also be used in Africa with strengthen more over hospitals technical capacity in intensive care before and after surgical therapy [16].

Our study has some limitations that should be noted. Due to inadequacy of technical training in regional hospitals, IS surveillance is ongoing only in national referral hospitals located at the capital city. This limitation is related to a lack of radiological technology system, especially in regional hospitals but also at national referral hospitals. Enema reduction was not performed in children, because of lack of adequate contrast medium for this age even at the national referral hospitals. Ultrasound is performed only at national referral hospitals and pediatric surgeons are not available at regional hospitals. Some infants with IS may died, especially in rural zones far from the capital city, without any identification of the cause of the death. Another limitation of the study is the small population of Togo. It does not allow an evaluation of association between IS and rotavirus vaccine at the local level.

## Conclusion

This active surveillance for IS in Togo has outlined the exclusive use of surgery as IS therapy method, often associated to an intestinal resection with a very high case fatality rate. Continued enhanced surveillance is needed to identify risk factors for intestinal resection and death.

### What is known about this topic

- Delay in seeking care, intestinal resection is common, and the case fatality rate is high.

### What this study adds

- No significant seasonality, children in 5-7 age-group are more affected, and surgery is the only method of treatment.
